# Detection of genes responsible for cetuximab sensitization in colorectal cancer cells using CRISPR-Cas9

**DOI:** 10.1042/BSR20201125

**Published:** 2020-10-21

**Authors:** Ting-ting Hu, Jia-wen Yang, Ye Yan, Ying-ying Chen, Hai-bo Xue, You-qun Xiang, Le-chi Ye

**Affiliations:** 1 Department of Gastroenterology and Hepatology, The First Affiliated Hospital of Wenzhou Medical University, Wenzhou 325003, P.R. China; 2 Department of Colorectal and Anal Surgery, The First Affiliated Hospital of Wenzhou Medical University, Wenzhou 325003, P.R. China; 3 Zhejiang Clinical Research Center of Minimally Invasive Diagnosis and Treatment of Abdominal Diseases, Wenzhou 325000, P.R. China

**Keywords:** cetuximab, clustered regularly interspaced short palindromic repeats (CRISPR)-Cas9, Colorectal cancer, drug sensitivity

## Abstract

Colorectal cancer (CRC) is a common malignant tumor in digestive tract with highly invasive and metastatic capacity. Drug sensitivity remains a significant obstacle to successful chemotherapy in CRC patients. The present study aimed to explore genes related to cetuximab (CTX) sensitivity in CRC by clustered regularly interspaced short palindromic repeats (CRISPR)-Cas9. Celigo image cytometer was used to detect suitable cells and optimal dosage of CTX. Inhibition rate of CTX on Caco-2 cells was evaluated by cell counting kit-8 (CCK-8) method before and after transfection. 3-(4,5-dimethylthiazol-2-yl)2,5-diphenyl tetrazolium bromide (MTT) was performed to explore suitable concentration of puromycin and multiplicity of infection (MOI). CRISPR-Cas9, sequencing data quality analysis and cell viability test were used for the selection of genes related to CTX sensitivity in CRC cells. Finally, the selected genes associated with CTX sensitivity in CRC cells were further validated by colony formation and CCK-8 assays. In the present study, Caco-2 cells had a better prolificacy, and CTX 100 μg/ml exhibited a good inhibition trend on the 7th and 14th days of infection. MTT assay indicated that the minimum lethal concentration of puromycin was 2.5 μg/ml. Forty-six candidate genes were preliminarily screened via sequencing data quality analysis. Subsequently, we found that knockout of any of the four genes (*MMP15, MRPL48, CALN1* and *HADHB*) could enhance CTX sensitivity in Caco-2 cells, which was further confirmed by colony formation assay. In summary, *MMP15, MRPL48, CALN1* and *HADHB* genes are related to the mediation of CTX sensitivity in CRC.

## Introduction

Colorectal cancer (CRC) is a highly invasive and metastatic malignant tumor of the digestive tract, with high mortality [1]. Most CRC cells are currently assumed to be stem cells or stem cell-like cells. These stem cells are the outcomes of progressive accumulation of genetic and epigenetic changes that activate oncogenes and inactivate tumor-suppressor genes [2]. Due to the low early diagnosis rate, most patients are diagnosed as middle or advanced CRC, resulting in difficult treatment and poor prognosis [3]. At present, chemotherapy and radiotherapy are typical therapies for CRC. Systemic treatment of metastatic CRC usually involves chemotherapy backbone (fluoropyrimidines, oxaliplatin and irinotecan chemotherapies form) combined with biotherapy [4]. However, despite therapeutic advancements, the prognosis for metastatic CRC patients remains poor, with a median overall survival of 18–21 months [5]. Drug resistance is the main reason for the failure of chemotherapy in cancers.

Apart from chemotherapy and radiotherapy, advances have been achieved by the addition of targeted agents. Cetuximab (CTX) (Erbitux) is a monoclonal antibody that targets epidermal growth factor receptor to impede the phosphorylation of receptor-associated kinases and related signaling cascade [6], and plays an anti-tumor role at both cellular and genetic levels [7]. Cetuximab was also demonstrated to enhance overall survival when combined with radiotherapy alone [8]. Segelov et al. have clarified that the median survival of advanced CRC patients can be extended from 6–7 to 24–30 months after treatment with combined targeted drugs such as CTX and chemotherapy [9]. However, after a period of CTX treatment, numerous patients become insensitive to CTX with disease progression. CTX can kill cancer cells that are sensitive to drugs, but the remaining resistant cells can cause tumor recurrence and even metastasis. Currently, the vital challenge for CRC treatment is to improve sensitivity to CTX. Therefore, it is of great significance to find new CTX-sensitized targets in CRC.

Clustered regularly interspaced short palindromic repeats (CRISPR)-Cas9 is a special structure found in the genomes of bacteria and archaea that protect them from phages and plasmids [10]. Scientists have transformed CRISPR-Cas9 into a simple, efficient and accurate genome editing tool, providing an effective method for finding functional genes [11]. For CRC, the use of CRISPR-Cas9 to explore genes related to CTX sensitivity has not been reported. Therefore, in the present study, we screened novel genes involved in CTX sensitivity in CRC cells based on genome-scale CRISPR-Cas9 knockout (GeCKO) screening and coupled with cell viability test.

## Materials and methods

### Cell culture

HT-29 and Caco-2 cells were obtained from the Cell Biology Institute of Chinese Academy of Sciences. The cells were cultured in DMEM (Gibco, Invitrogen, U.S.A.), supplemented with 10% fetal bovine serum (HyClone Laboratories, Logan, UT), 100 U/ml penicillin and 100 µg/ml streptomycin (Invitrogen, CA, U.S.A.) at 37°C in a humidified atmosphere of 5% CO_2_.

### CRISPR-Cas9 knockout screening

To identify genes associated with enhanced sensitivity of CRC to CTX, GeCKO screening with a pooled GeCKO library was performed. We applied a GeCKO library of 123411 single-guide RNAs (sgRNAs) targeting 20914 human genes, including 19050 annotated protein-coding genes and 1864 microRNA expression genes, with an average coverage of six sgRNAs per gene. A single lentiviral vector system (lentiCRISPRv2) of GeCKO library was used to transfect HT-29 and Caco-2 cells at a low multiplicity of infection (MOI) to ensure that most cells gained only one viral construct. After 72 h of transduction, HT-29 and Caco-2 cells were screened with puromycin, and the cells that survived after 7 days were successfully transfected cells. A total of 3 × 10^7^ cells were directly harvested as day 0 group after puromycin selection. Then the remaining cells were treated with dimethyl sulfoxide (DMSO) vehicle and 100 μg/ml CTX for 7 and 14 days, respectively.

### Cell growth curve assay

HT-29 and Caco-2 cells were plated in triplicate in 96-well plates at a density of 2000 cells per well. After culturing at 37°C in a 5% CO_2_ incubator for another day, Celigo image cytometer (Cyntellect Inc., CA, U.S.A.) was used to capture cell images once a day for 4 days. Cell numbers in each well were quantified by Celigo image cytometer, and cell growth curves were generated for each cell.

### MTT assay

To explore the most suitable concentration of puromycin, 3-(4,5-dimethylthiazol-2-yl)2,5-diphenyl tetrazolium bromide (MTT) (Sigma Chemical Company, MO, U.S.A.) was used to assess the proliferation of Caco-2 cells. Briefly, Caco-2 cells were seeded in 96-well plates in triplicate at the density of 2000 cells per well, followed by addition of puromycin at various concentrations (0, 1.25, 2.5, 5, 10 μg/ml). Then the culture medium was discarded, and 20 µl of MTT (5 mg/ml) were added into each well. After 7 days of incubation, the MTT crystals were dissolved in DMSO and the absorbance at 490 nm was measured.

### Cell proliferation analysis via Celigo image cytometer

To find out the best dosage of CTX, Celigo image cytometer was used to quantify the cell number. The Caco-2 cells were placed in 96-well plates in triplicate and then adding CTX at a range of concentrations (0, 50, 100, 200, 400, 600, 800, 1200 μg/ml). After culturing at 37°C in a 5% CO_2_ incubator for another day, Celigo image cytometer was used to capture cell images for 14 days. Cell numbers in each well were quantified by Celigo image cytometer, and cell growth curves were generated for each cell.

### Determination of optimal MOI

Caco-2 cells with and without puromycin were divided into group A and group B, respectively. Cells were infected with different MOI values (0.25, 0.5, 1, 2, 10) and seeded in 96-well plates at the density of 6000 cells per well. Puromycin (2.5 μg/ml) was then added to each well in group A only. Forty-eight hours later, the culture medium was discarded, and 20 µl of MTT (5 mg/ml) were added to each well. Following a 4-h incubation, the crystals were dissolved by DMSO and the absorbance at 490 nm was read. The functional MOI was defined as the MTT value of group A/the MTT value of group B [12].

### Sequencing data quality analysis

NEBNext® Ultra™ DNA Library Prep Kit for Illumina® kit (QIAGEN Company, Hilden, Germany) was used for the construction of library. Briefly, more than 50 ng PCR products purified by gel cut or magnetic beads were adopted to construct the library directly. End Prep Enzyme Mix was used for end repair, including 5′-end phosphorylation and 3′-end of ‘A’ plus, with addition of sequencing adapter at both ends, followed by purification via AxyPrep Mag PCR Clean-up. Finally, P5 and P7 primers were used for amplification, in which the index containing 6-bp base at the end of P7 was used for subsequent sample discrimination. After purification, the quality and concentration of library were measured by Agilent 2100 bioanalyzer (Agilent Technologies, CA, U.S.A.) and Qubit 2.0 fluorophotometer (Invitrogen, CA, U.S.A.), respectively. Subsequently, 2× 150 bp paired-end sequencing (PE) was performed according to Illumina HiSeq instruction (Illumina, CA, U.S.A.), and the sequence information was read by HiSeq Control Software (HCS) + OLB + GAPipeline-1.6.

### Cell viability test

The infected cells in logarithmic phase were seeded in 96-well plates and were treated with 100 μl of CTX 100 μg/ml or DMSO, respectively. Meanwhile, normal control (NC) group was set up and treated with CTX 100 μg/ml or DMSO, respectively. Five days later, 100 μl of CellTiter-Glo (thawed 24 h at 4°C in advance) was added to each well and shook on an oscillated instrument for 10 min to induce cell lysis. Subsequently, an enzyme mark instrument was adopted to measure the absorbance of each well in Luminescent mode. The PC interference background, multiples of inhibited proliferation and sensitization multiples of each group of cells were calculated by absorbance of each well.

Day 5 proliferation multiples = Cell number of day 5/Cell number day 1
PC background interference = Day 5 proliferation multiples of infected cell treated with DMSO/Day 5 proliferation multiples of normal cell treated with DMSO
Multiples of inhibited proliferation = Day 5 proliferation multiples of infected cell treated with DMSO/Day 5 proliferation multiples of infected cell treated with CTX
Sensitization multiples = Multiples of inhibited proliferation of infected cell/Multiples of inhibited proliferation of normal cell

### Experimental grouping

To evaluate the genes that were related to the CTX sensitivity in CRC cells, cell viability, cell growth and colony formation assays were performed. Caco-2 cells were divided into ten groups, namely, NC group (DMSO), NC+CTX 100 μg/ml group, MMP15 knockout group (DMSO), MMP15 knockout + CTX 100 μg/ml group, MRPL48 knockout group (DMSO), MRPL48 knockout + CTX 100 μg/ml group, CALN1 knockout group (DMSO), CALN1 knockout + CTX 100 μg/ml group, HADHB knockout group (DMSO) and HADHB knockout + CTX 100 μg/ml group.

### Cell counting kit-8 assay

After transfection, Caco-2 cells were seeded in 96-well plates in triplicate at the density of 2 × 10^3^ cells per well, followed by addition of CTX 100 μg/ml. After culturing for 24, 48, 72, 96 and 120 h, cell counting kit-8 (CCK-8) solution 100 μl (Sigma) was added into each well, and plates were incubated at 37°C for 1 h. Microplate reader was used to test optical density at the wavelength of 450 nm.

### Colony formation assay

Colony formation assay was adopted to evaluate the effects of Caco-2 cells proliferation *in vitro*. Cells were plated in a six-well plate containing 1 × 10^3^ cells per well in triplicate. After 2 weeks, the cells were fixed with 4% paraformaldehyde for 15 min and stained with 0.1% Crystal Violet. The visible colonies were counted.

### Statistical analysis

Statistical analysis was performed by SPSS (version 20.0) and the experimental measurement data were expressed as mean ± SD. Statistical differences between groups were determined by one-way or two-way ANOVA. Differences were considered significant when *P*<0.05.

## Results

### Growth curve results

**Figure 1:**
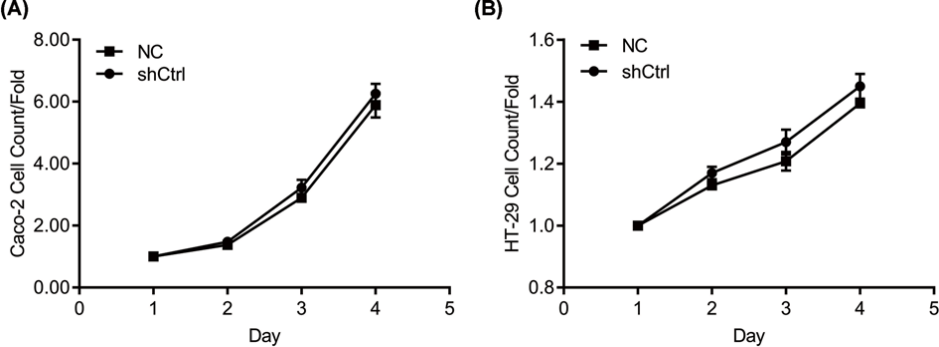
Caco-2 cells had a better prolificacy Proliferation multiples of the infected (**A**) Caco-2 and (**B**) HT-29 cells.

From Figure 1A,B, the proliferation multiples of Caco-2 and HT-29 cells significantly increased in the first 4 days. However, on the 3rd day, the proliferation multiples of Caco-2 cells were higher than 2, while that of HT-29 cells was lower than 2, indicating that infected Caco-2 cells had a better prolificacy. Therefore, Caco-2 cells were selected for the following experiments.

### Optimal concentration of puromycin

**Figure 2:**
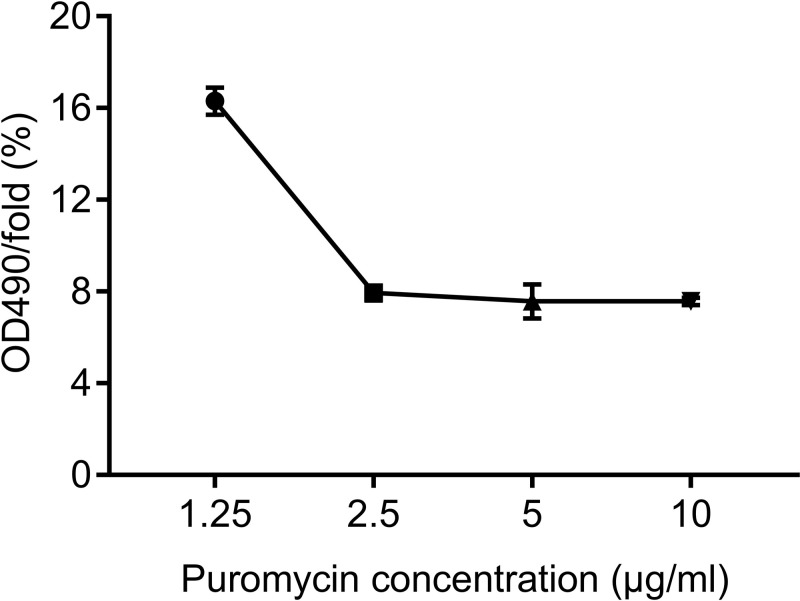
The OD value of Caco-2 cells treated with puromycin at different concentrations (0, 1.25, 2.5, 5, 10 μg/ml)

MTT assay results revealed that the percentage of viable Caco-2 cells treated with 1.25, 2.5, 5 and 10 μg/ml puromycin for 2 days were 16.3, 7.9, 7.6 and 7.6%, respectively. Therefore, the optimal lethal concentration of puromycin was 2.5 μg/ml (Figure 2).

### Optimal dosage of CTX

**Figure 3:**
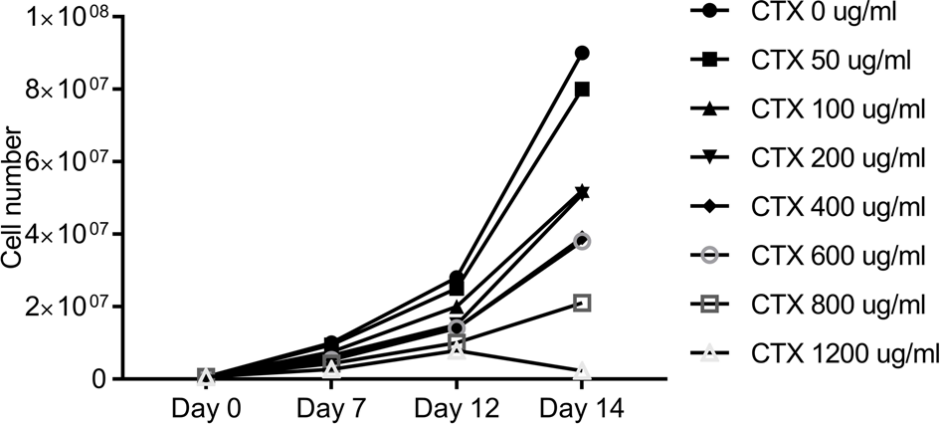
The number of Caco-2 cells after CTX treatment at different concentrations (0, 50, 100, 200, 400, 600, 800, 1200 μg/ml)

As shown in Figure 3, CTX suppressed Caco-2 cell proliferation in a dose-dependent manner, and the IC_50_ of CTX was 245.7 μg/ml. On the 14th day, when the CTX concentration was 50, 100, 200, 400, 600, 800 and 1200 μg/ml, the inhibitory rate was 11.67, 42.33, 43.67, 56.33, 57.67, 76.67 and 97.46%, respectively, indicating that CTX had a significant inhibitory function on Caco-2 cells in a dose-dependent manner. In the case that three CTX concentrations of 100, 200, 400 μg/ml had similar inhibitory effect and considering the high cost of CTX, CTX 100 μg/ml was selected as the optimal concentration for subsequent experiments.

### Optimal MOI results

After 48 h of infection with Caco-2 cells, we found that the fluorescence rate augmented with the increase in infection intensity in group A, but not in group B. When MOI was 0.5, the fluorescence rate was 43% in group A, and 86% in group B, with 50% Functional MOI. MOI = 0.5 was selected for the subsequent experiments (Table 1).

**Table 1:** The effect of different MOI on Functional MOI

Cell number/well	MOI	MTT test value of group B	MTT test value of group A	Functional MOI
6000	10	0.60	0.44	0.74
6000	2	0.57	0.40	0.69
6000	1	0.89	0.44	0.50
6000	0.5	0.86	0.43	0.50
6000	0.25	0.97	0.28	0.29

### Sequencing data quality analysis results

Sequencing data quality analysis was used to screen genes meeting the screening criteria (|FC Caco2| < 2, FCERBITUX ≤ −2; BF Caco2 < 0, BFERBITUX > 2) between Day 7 vs Day 0 and Day 14 vs Day 0. A total of 16 genes were obtained from the intersection in accordance with 209 genes acquired from the preliminary selection of Day 7 vs Day 0 and 307 genes obtained from the preliminary selection of Day 14 vs Day 0. In addition, another 30 genes were selected according to the screening criteria (day 14 ERBITUX logFC was sorted from small to large, Cacao2 logFC was less than 0.5) (Table 2). However, previous report has reported that *BCR* and *GEM* genes are closely related to CTX sensitivity, so we only focused on the remaining 44 genes [13].

**Table 2:** Candidate genes screened by sequencing data quality analysis

Entrez ID	Entry	Caco2 logFC	ERBITUX logFC
The intersecting genes of Day 7 vs Day 0 and Day 14 vs Day 0			
83698	CALN1	−0.151	−2.598
1690	COCH	0.244	−2.701
2824	GPM6B	−0.041	−1.119
25960	GPR124	0.137	−1.054
4324	MMP15	−0.630	−3.279
79570	NKAIN1	−0.232	−2.910
403277	OR5K3	0.340	−1.837
340990	OTOG	−0.289	−3.768
29944	PNMA3	0.072	−2.672
5706	PSMC6	−0.344	−1.244
29127	RACGAP1	0.137	−5.228
2030	SLC29A1	−0.141	−1.166
84851	TRIM52	0.264	−1.300
51322	WAC	−0.741	−3.744
56949	XAB2	−0.556	−2.979
80032	ZNF556	−0.433	−4.791
The genes that met the screening criteria (screening criteria: day 14 ERBITUX logFC was sorted from small to large, Caco2 logFC was less than 0.5)			
4897	NRCAM	−0.243	−5.262
3554	IL1R1	0.053	−5.206
402415	XKRX	−0.030	−5.132
613	BCR	0.041	−4.895
51642	MRPL48	−0.102	−4.776
5422	POLA1	−0.172	−4.679
7579	ZSCAN20	−0.164	−3.717
84455	EFCAB7	−0.051	−3.718
90522	YIF1B	0.218	−3.371
85480	TSLP	0.071	−3.486
79065	ATG9A	−0.203	−3.243
130497	OSR1	−0.223	−3.196
200132	TCTEX1D1	−0.231	−3.177
64221	ROBO3	−0.188	−3.206
92421	CHMP4C	−0.194	−3.155
1373	CPS1	0.013	−3.309
79083	MLPH	0.105	−3.114
23589	CARHSP1	−0.037	−3.149
3646	EIF3E	0.165	−2.956
2734	GLG1	−0.128	−2.984
3032	HADHB	0.182	−2.920
2669	GEM	−0.198	−2.900
58524	DMRT3	0.176	−2.873
1608	DGKG	−0.195	−2.814
51116	MRPS2	−0.241	−3.365
84530	SRRM4	0.228	−3.255
138882	OR1N2	0.192	−3.145
161424	NOP9−	−0.185	−3.106
5588	PRKCQ	−0.227	−2.856
4762	NEUROG1	0.012	−2.999

### Cell viability by CellTiter-Glo luminescence assay

To measure the PC background interference, multiples of inhibited proliferation and sensitization multiples of the 44 genes, CellTiter-Glo luminescence assay was used to determine the proliferation of cells. Genes with PC background interference multiples between 0.7 and 1 (closer to 1 indicated less background interference), multiples of inhibited proliferation greater than 1.5, and sensitization multiples greater than 1.2 were selected. There were four genes meeting the selection criteria, namely *MMP15, MRPL48, CALN1* and *HADHB* (Table 3).

**Table 3:** Cell viability test results of cells with different genes knocked out

Target	Day 5 proliferation multiple	PC background interference	Proliferation inhibition multiple	Sensitizing multiple
NC	10.43	1.00	1.31	1.00
NC + CTX	7.94			
MMP15	7.48	0.72	1.56	1.19
MMP15 + CTX	4.80			
MRPL48	7.28	0.70	1.61	1.23
MRPL48 + CTX	4.51			
CALN1	7.69	0.74	1.69	1.29
CALN1 + CTX	4.54			
HADHB	8.33	0.80	1.71	1.30
HADHB + CTX	4.88			

### CTX combined with genes knockout suppressed CRC cell viability and proliferation

**Figure 4:**
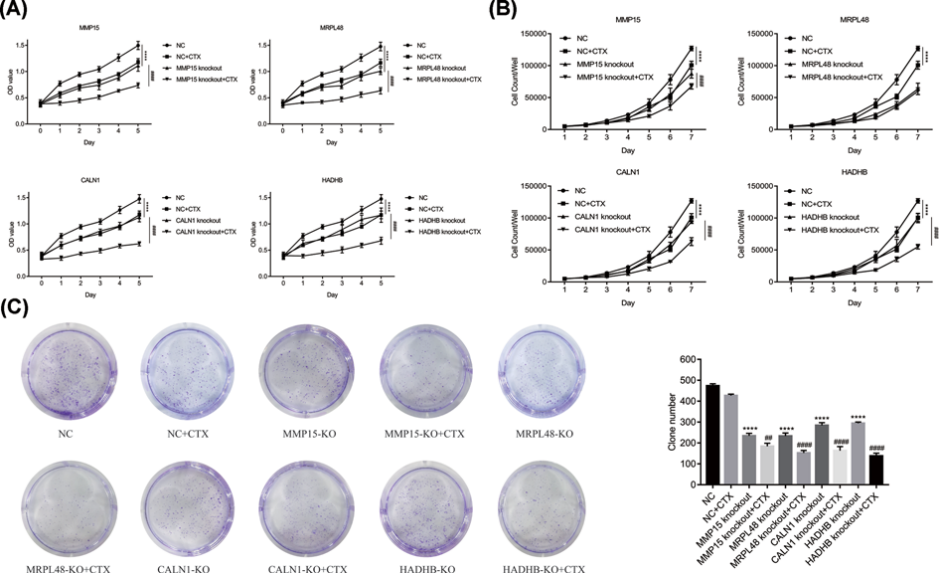
Caco-2 cells were divided into NC group, NC + CTX 100 μg/ml group, MMP15 knockout group, MMP15 knockout + CTX 100 μg/ml group, MRPL48 knockout group, MRPL48 knockout + CTX 100 μg/ml group, CALN1 knockout group, CALN1 knockout +CTX 100 μg/ml group, HADHB knockout group and HADHB knockout + CTX 100 μg/ml group CCK-8 assays were used to determine the (**A**) viability and (**B**) proliferation of Caco-2 cells. (**C**) Colony formation assay showed that CTX 100 μg/ml combined with knockout of MMP15, MRPL48, CALN1 and HADHB genes impaired the colony formation capacity of Caco-2 cells. ****P<0.0001 vs NC group; ^##^*P*<0.01, ^####^*P*<0.0001 vs gene knockout alone group.

From Figure 4A,B, knockout of *MMP15, MRPL48, CALN1* and *HADHB* genes suppressed Caco-2 cell viability and proliferation. On day 7, the inhibitory rate of Caco-2 cells in MMP15 knockout + CTX 100 μg/ml, MRPL48 knockout + CTX 100 μg/ml, CALN1 knockout + CTX 100 μg/ml and HADHB knockout + CTX 100 μg/ml groups were 46.8, 52.68, 50.26 and 56.61, respectively, which were significantly higher than that in NC + CTX 100 μg/ml group (20.64%). These findings revealed that *MMP15, MRPL48, CALN1* and *HADHB* genes knockout might facilitate the sensitivity of CRC cell line Caco-2 to CTX.

### Clonal formation results

To verify the sensitization effect of the above four gene knockout and evaluate its long-term sensitization effect, the clonal formation ability of Caco-2 cells was further tested through cell cloning experiment. As shown in Figure 4C, *MMP15, MRPL48, CALN1* and *HADHB* genes knockout alone reduced the cloning potential of Caco-2 cells. On this basis, the addition of CTX 100 μg/ml inhibited the clonal formation of Caco-2 cells more significantly than the gene knockout alone group (*P*<0.0001).

## Discussion

In the present study, GeCKO screening and cell viability test results revealed that *MMP15, MRPL48, CALN1* and *HADHB* genes might be involved in drug sensitivity to CTX in CRC cell (Caco-2). This finding suggested that *MMP15, MRPL48, CALN1* and *HADHB* genes were potential targets for the treatment of CRC, which fills in the gap of genes responsible for CTX sensitivity in CRC.

CRISPR-Cas9 forms a complex by using nuclease Cas9 protein and sgRNA [14]. To our knowledge, sgRNA determines the specificity of target sequence through base complementary pairing; Cas9 protein acts as nuclease to cut genomic DNA complementary to the spacers on the sgRNA, causing double-stranded DNA damage, thereby introducing genetic mutations through mechanisms [15]. As the third generation of gene editing technology, CRISPR-Cas9 has attracted wide attention due to its advantages of simple operation, flexible design, low price and multi-point editing. With the continuous development and improvement of CRISPR-Cas9 system, a series of drug-resistance genes or virus-resistance genes have been screened [16]. Chen and Zhang have found that CRISPR-Cas9 can revert resistance gene mutations [17]. Zhang et al. also believed that CRISPR-Cas9 could be used to construct drug-resistant cancer cell lines for drug screening [18]. In this study, we preliminarily obtained 44 candidate genes in Caco-2 CRC cells related to CTX resistance by CRISPR-Cas9. Subsequently, based on the selection criteria of PC background interference multiples between 0.7 and 1, multiples of inhibited proliferation greater than 1.5, and sensitization multiples greater than 1.2, four genes including *MMP15, MRPL48, CALN1* and *HADHB* were selected via CellTiter-Glo luminescence assay.

Metalloproteinases (MMPs) is a family of proteolytic enzymes that can maintain the dynamic equilibrium of extracellular matrix by degrading and remodeling extracellular matrix [19]. Several studies have shown that MMPs are related to inflammation, atherosclerosis, liver cirrhosis, connective tissue disease and cancer [20,21]. As the active center of mitochondrial ribosomes, mitochondrial ribosomal proteins (MRPs) are the basis for the normal expression of mitochondrial DNA and responsible for translation of encoding proteins of mitochondrial DNA [22]. Wang et al. have noticed that down-regulation of *MRPL18* gene expression can cause metabolic disorders in mouse insulinoma cells (MIN6) [23]. It is well known that changes in the concentration and activity of intracellular Ca^2+^ have a certain effect on the generation of drug resistance in tumors [24]. Human *CALN1* gene is only expressed in the brain, especially in the hippocampus and cerebral cortex. Sequence alignment done by Wu et al. has showed that *CALN1* gene is highly similar to the ubiquitous calcium mediator protein, calmodulin [25]. Calmodulin is the principle mediator of the Ca^2+^ signal found in many eukaryotic cells [26]. The proliferation, migration and metastasis of tumor cells require a large amount of energy, which means they need a more vigorous metabolism than normal cells. In addition to sugars that provide energy for the active proliferation of tumor cells, fatty acids can also produce ATP through β-oxidation [27]. *HADHB* mainly participates in the last three steps of fatty acid β-oxidation, including hydration of 2-dienoyl-CoA, dehydrogenation of fatty acid-2-hydroxyacyl-CoA, and thiolysis of β-ketoacyl CoA [28]. With a 3-year follow-up of 91 patients diagnosed with hilar cholangiocarcinom, Zhang et al. have found that *HADHB* not only shows high expression in hilar cholangiocarcinom, but also have a close relationship with tumor infiltration and lymphatic metastasis [29]. Nevertheless, no studies have shown the relationship between *MMP15, MRPL48, CALN1* and *HADHB* genes and CTX sensitivity.

On the basis of our prophase research, we speculated that *MMP15, MRPL48, CALN1* and *HADHB* genes may be related to CTX sensitivity in CRC. To verify the idea, CCK-8 assay was performed on cells that had *MMP15, MRPL48, CALN1* and *HADHB* genes knocked out. Results showed that *MMP15, MRPL48, CALN1* and *HADHB* genes knockout alone remarkably suppresed viability and proliferation of Caco-2 cells, and the addition of CTX 100 μg/ml inhibited cell viability and proliferation more significantly than the gene knockout alone group. Moreover, the sensitization effect of the above four gene knockout was further confirmed by clonal formation assay. Taken together, these data revealed that *MMP15, MRPL48, CALN1* and *HADHB* genes knockout might facilitate the sensitivity of CRC cell line Caco-2 to CTX.

In summary, the present study confirms that *MMP15, MRPL48, CALN1* and *HADHB* genes are responsible for CTX sensitivity in CRC, which is the first time to explore genes related to CTX sensitivity by CRISPR-Cas9, providing a theoretical basis for further research on drug-sensitivity mechanism of CRC.

## Abbreviations

CCK-8: cell counting kit-8
CRC: colorectal cancer
CRISPR: clustered regularly interspaced short palindromic repeats
CTX: cetuximab (Erbitux)
DMSO: dimethyl sulfoxide
GeCKO: genome-scale CRISPR-Cas9 knockout
MOI: multiplicity of infection
MTT: 3-(4,5-dimethylthiazol-2-yl)2,5-diphenyl tetrazolium bromide
NC: normal control
sgRNA: single-guide RNA
